# A survey on patients’ knowledge and expectations during informed consent for spinal surgery: can we improve the shared decision-making process?

**DOI:** 10.1186/s13037-016-0103-z

**Published:** 2016-06-03

**Authors:** Sebastian Weckbach, Tugrul Kocak, Heiko Reichel, Friederike Lattig

**Affiliations:** Department of Orthopedics, University of Ulm, Oberer Eselsberg 45, 89081 Ulm, Germany; Berit Paracelsus Klinik AG, Steinweg 1, CH 8052 Niederteufen, Switzerland

**Keywords:** Informed consent, Spinal surgery, Safety in surgery

## Abstract

**Background:**

The informed medical consent in surgery requires to some point basic medical knowledge. The treating physicians while explaining the details and risks of the recommended procedure often imply this. We hypothesized, that patients do not have adequate medical understanding to decide about the ongoing therapy and its potential complications based on knowledge jeopardizing the patients’ safety.

**Methods:**

We conducted a retrospective analysis of a prospective database using a multiple choice questionnaire with 10 basic questions about anatomy, clinical symptoms and therapies of spinal diseases in our spine clinic at a German university hospital. Included were all patients at the spine clinic who agreed to the study and to fill in the questionnaire. Furthermore the patients age, mother tongue, the past spinal surgical history, the length of duration of symptoms and the patients education were inquired. The data were analyzed descriptive.

**Results:**

Included were 248 patients with an average age of 59 years (16–88 a). 70 % of all patients used German as their mother tongue. 30 % of the included patients already had spinal surgery and suffered on average for 13.4 years because of their spinal disorder. Overall 32.6 % of all questions were answered correctly (range 0.8–68 %). A correlation of correctly answered questions and the patients’ age, duration of symptoms, mother tongue, education and past surgical history could not be described.

**Conclusion:**

The percentage of correctly answered questions is almost as low as the likelihood of nearness in guessing. Having this in mind the patients do not choose any treatment option based on knowledge. The physicians need to provide more basic knowledge to the patients. This would increase the amount of successful therapies, content patients and the patients safety.

**Electronic supplementary material:**

The online version of this article (doi:10.1186/s13037-016-0103-z) contains supplementary material, which is available to authorized users.

## Background

All surgical procedures require a written informed medical consent to present the expected outcome, therapeutic alternatives, the procedure associated specific potential complications as well as to meet legal aspects. To understand the complexity of surgical procedures is of great importance [1, 2]. With the implicit understanding that patients have basic medical knowledge physicians explain the procedures to help the patients to take the decision made on information and knowledge and actively participate in their treatment. Adequate patients education is significant for the patients satisfaction after surgery [3]. Failure in patients understanding is also a potential safety issue [4].

We hypothesized that the patients do not have the medical education to decide on their treatment based on knowledge and information.

## Methods

We conducted a retrospective analysis of a prospective database using a multiple choice questionnaire with 10 basic questions about anatomy, clinical symptoms and therapies of spinal diseases in our spine clinic at a German university hospital from 01/01/2013 to 06/01/2013. Included were all adult patients of our special spine clinic (range 18–88 a) willing to fill in the multiple-choice questionnaire and signing consent for this study. Furthermore the patients’ age, mother tongue, past spinal surgical history, the duration of symptoms, the profession and the educations were assessed. The multiple-choice questionnaire consisted of 10 questions and is shown in (Additional file 1) in detail. Outcome parameters were the number of correctly answered questions. Anymore we were analyzing if there is a correlation between correctly answered questions and the patients age, the education, the profession, the past spinal surgical history, the duration of symptoms or the patients mother tongue. The study protocol fulfilled the requirements by the university care committee Tuebingen, Germany. The statistics were descriptive and results presented in percent of all answered questions.

## Results

**Fig. 1:**
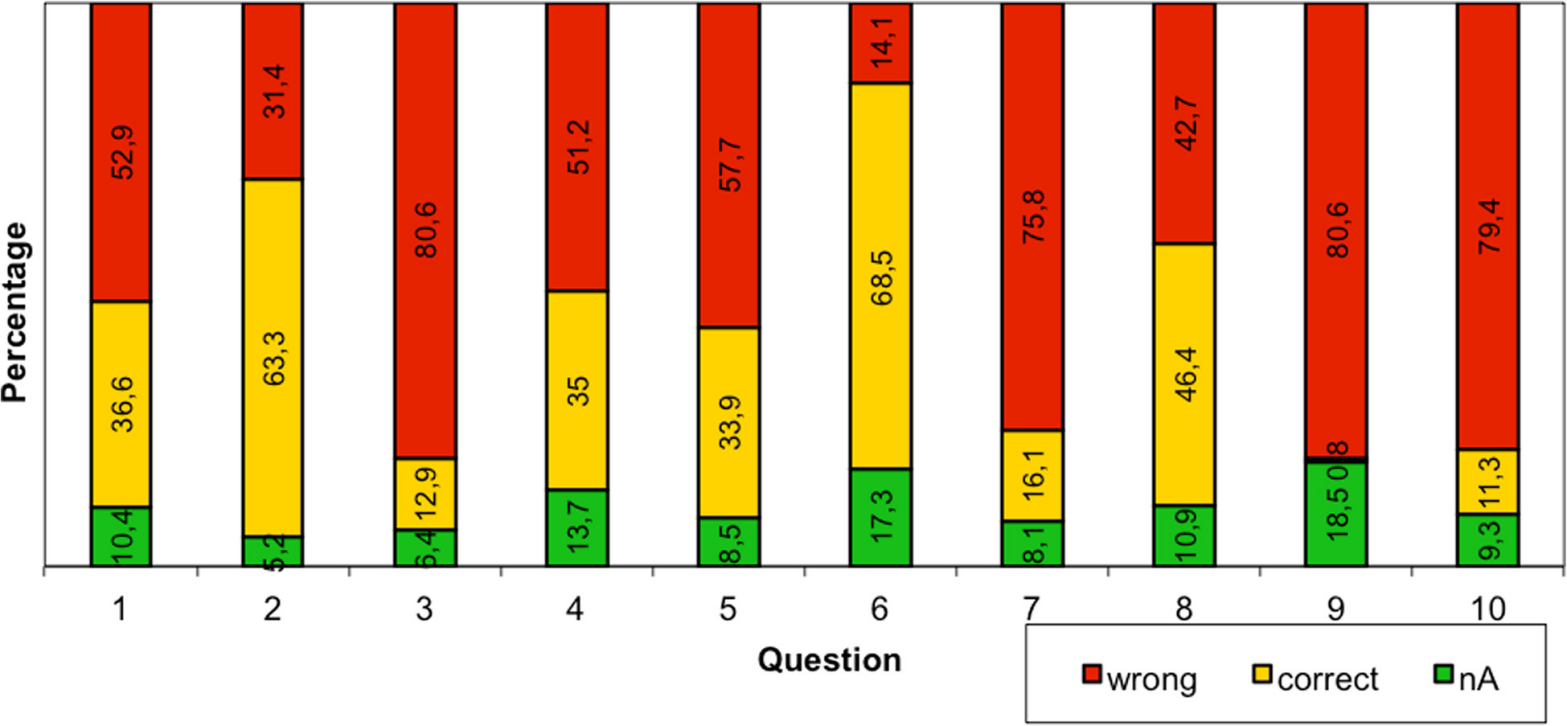
Results of the questionnaire subdivided into the different questions and its percentage of correctly, wrong and not answered queries

During the above-mentioned study period 248 patients could be included (participation 50 %). The mean age of all participants was 58.5 years (range 18–88 years). 70 % of the patients declared German to be their mother tongue. The patients complained about having pain for 13.4 years in average. 30 % of all included patients had already spinal surgery. Question number 1 answered just 37 % correctly, 10 % did not answer at all and 53 % gave the wrong answer. 64 % of the participants marked question 2 correctly, 32 % wrongly and 5 % did not answer. Question number 3 was in 81 % not correct, 13 % knew the solution and 6 % did not respond. Question 4 showed the following results: 14 % no answer, 35 % correct, 51 % wrong. 8.5 % did not specify question 5, whereas it was correctly done by 34 % and 58 % were mistaken. 69 % of all participants answered question 6 correctly, which was the best results within this study. 17 % did not give an opinion on it and 14 % were wrong. Question 7 was answered as followed: 16 % correct, 76 % wrong, 8 % no answer. Similar results could be found in question 8: 46 % correct, 43 % wrong, 11 % no answer. Just 1 % of the study participants knew the solution to question 9, 19 % did not answer and 81 % responded wrongly. Likewise question 10: 11 % correct, 80 % wrong, 9 % no answer. Overall just 32.6 % of all questions were answered correctly. All results are shown in Fig. 1.

Furthermore the correlation between correctly answered questions and the patients age, mother tongue, period of duration of symptoms as well as the educative background were investigated. No positive correlation could be found between all subgroups and the correctly answered questions (Data not shown).

## Discussion

Informed medical consent is crucial and goes far beyond signing a form [5]. Nowadays this is put more to the physicians’ attention because of a dramatic emergence in medico legal processes [6]. Joolaee S. et al. reported that 48 % of the patients did not even read the form before signing [7]. Consent is thought to be a “ritualistic legal procedure” and not a basis for the patients needs to decide on the procedure [8, 9]. To improve the patients safety adequate knowledge is an important prerequisite for the patient-centered medicine [10, 11]. However, not just in surgery but across many medical subspecialties the patients’ expectations were not met [12–14]. Rothberg et al. published recently that 88 % of the patients were mistaken about the expectations of a cardiologic intervention [15]. Severe effort was already put into improvement of patient comprehension in informed consent [16]. According to Schenker et al. three practical issues –“More is not always better”, “Timing Matters”, “Technology can help”- should be taken into consideration [17].

The above-mentioned findings of a dramatic lack in patients understanding of an informed medical consent is strongly supported by our presented data. Akkad et al. reported a higher satisfaction if the consent was read and understood by the patients [18]. However various reasons for a current bad practice in consenting is found on the patients side due to a lack in basic knowledge but also due to a bad practice in decision making by the surgeons [19]. The patients were given in mean 23.1 s to make their statement [20].

Interestingly basic knowledge is not depending on the patients’ age, previous surgical history, the duration of symptoms, the patients’ mother tongue and the educational background. This is in contrast to Paasche-Orlow et al., who reported a lack of health literacy which is associated with education, ethnicity and age [21]. Besides this, the patients literacy abilities are overestimated by physician [22]. This stops the patients from gathering more details of the treatment plan [23].

This study highlights the lack of educated informed consent in surgery clearly. Regarding our data, more basic medical knowledge is to be provided to reach the patients and the surgeons’ goals. It demonstrates that all for the patients available sources such as daily press, physicians, internet and so on are not sufficient to provide even anatomic basics for the affected and therefore most interested area.

The study is prospective, but the period and the included amount of patients are not very high. Nevertheless this survey supports our hypothesis of a lack of knowledge in an educated informed consent for surgery and points out the necessity for further research and improvement in consenting for surgery.

## Conclusion

The patients’ informed consent is barely based on knowledge. Therefore some other effects like sympathy to the treating surgeon or the lack of alternative health care providers might influence the patients’ decision in agreeing to a certain surgical procedure. Obviously this condition can be merely accepted especially in high-risk surgical fields like spine surgery. Regarding this study patients need to be given more basic information of medical backgrounds to base their decisions on knowledge. Finally the surgeons need to improve their skills while educating the patient to increase the goals of their treatment plan, to increase the patients’ satisfaction as well as the patients safety.

## Additional file

Additional file 1: Questionnaire. (DOC 35 kb)
