# Supplementary material for: A survey on patients’ knowledge and expectations during informed consent for spinal surgery: can we improve the shared decision-making process?
Source: Patient Saf Surg. 2016 Jun 3;10:15. doi: 10.1186/s13037-016-0103-z (PMC4891841; doi:10.1186/s13037-016-0103-z)
Supplement: Additional file 1: — Questionnaire. (DOC 35 kb) [file 13037_2016_103_MOESM1_ESM.doc]

**Additional file 1**

**Dear patients,**

This short anonymous questionnaire is designed to analyze the patients’ knowledge about spinal diseases and its therapy in order to improve the patients’ informed consent. Please answer the following questions. With filling in this questionnaire you agree to the study. Thank you very much for your help.

Orthopedic Spine Clinic, University Ulm, Germany.

Personal information:

Age: (in years)

Mother tongue:

Gender: □ female □ male

Duration of symptoms? (In years)

Past spinal surgical history: □ yes □ no

Education: □ no degree □ high school □ university □ other

I am □ working □ retired

1. The human spine is consisting of how many vertebrae?

(Please mark only 1 answer)

□ 5 vertebrae

□ More than 5 vertebrae

□ More than 10 vertebrae

□ More than 15 vertebrae

□ More than 20 vertebrae

2. If you look at a human spine frontally it is regularly

(Please mark only 1 answer)

□ S-shaped

□ Straight

□ C-shaped

□ Double s-shaped

3. Looking at a human spine laterally it is

(Please mark only 1 answer)

□ S-shaped

□ Straight

□ C-shaped

□ Double s-shaped

4. Please order the parts of the human spine head to feet.

(Please number the parts as 1,2,3,4,5)

Sacrum ___

Thoracic spine ___

Cervical spine ___

Tailbone (coccyx) ___

Lumbar spine ___

5. The human spine is protecting what important structure(s)?

(Please mark, multiple answers are possible)

□ The aorta

□ The spinal cord

□ Nerves controlling the bladder

□ The portal vein

6. Does damage to the spinal cord at cervical spine level have different consequences to the patient than damage to mid thoracic spine?

□ Yes

□ No

7. What effects does a complete disrupture of the myelon mid thoracic level for the patient have?

(Please mark, multiple answers are possible)

□ Not being able to ambulate

□ Weakness in both arms

□ Sexual function disturbance

□ Incontinence

□ Disturbance of memory

□ Loss of temperature sensitivity in the legs

8. Can a distinct narrowing of the spinal canal (spinal canal stenosis) in the lumbar spine lead to neurologic issues in the arms?

□ Yes

□ No

9. Which statement(s) is/are correct?

(Please mark, multiple answers are possible)

□ The sciatic nerve is a bundle of multiple spinal nerves.

□ Damage to one spinal nerve can cause a complete paralysis of a leg

□ Spinal nerves only activate muscles

□ Spinal nerves quasi are the continuation of the myelon

□ The human body regularly consists of 31 spinal nerve pairs.

10. What statement(s) of the human intervertebral disc is/are correct?

(Please mark, multiple answers are possible)

□ The intervertebral disc connects two vertebrae.

□ The intervertebral disc consists of cartilage tissue.

□ The intervertebral disc consists of muscle tissue.

□ The myelon runs through the intervertebral disc.

□ The intervertebral disc serves as a buffer and allows movements between two vertebrae

**Thank you!**
